# Neural correlates of acoustic dissonance in music: The role of musicianship, schematic and veridical expectations

**DOI:** 10.1371/journal.pone.0260728

**Published:** 2021-12-01

**Authors:** Carlota Pagès-Portabella, Mila Bertolo, Juan M. Toro

**Affiliations:** 1 Center for Brain & Cognition, Universitat Pompeu Fabra, Barcelona, Catalunya, Spain; 2 Department of Psychology, Harvard University, Cambridge, Massachusetts, United States of America; 3 Institució Catalana de Recerca i Estudis Avançats (ICREA), Barcelona, Catalunya, Spain; University of Milano–Bicocca: Universita degli Studi di Milano-Bicocca, ITALY

## Abstract

In western music, harmonic expectations can be fulfilled or broken by unexpected chords. Musical irregularities in the absence of auditory deviance elicit well-studied neural responses (e.g. ERAN, P3, N5). These responses are sensitive to schematic expectations (induced by syntactic rules of chord succession) and veridical expectations about predictability (induced by experimental regularities). However, the cognitive and sensory contributions to these responses and their plasticity as a result of musical training remains under debate. In the present study, we explored whether the neural processing of pure acoustic violations is affected by schematic and veridical expectations. Moreover, we investigated whether these two factors interact with long-term musical training. In Experiment 1, we registered the ERPs elicited by dissonant clusters placed either at the middle or the ending position of chord cadences. In Experiment 2, we presented to the listeners with a high proportion of cadences ending in a dissonant chord. In both experiments, we compared the ERPs of musicians and non-musicians. Dissonant clusters elicited distinctive neural responses (an early negativity, the P3 and the N5). While the EN was not affected by syntactic rules, the P3a and P3b were larger for dissonant closures than for middle dissonant chords. Interestingly, these components were larger in musicians than in non-musicians, while the N5 was the opposite. Finally, the predictability of dissonant closures in our experiment did not modulate any of the ERPs. Our study suggests that, at early time windows, dissonance is processed based on acoustic deviance independently of syntactic rules. However, at longer latencies, listeners may be able to engage integration mechanisms and further processes of attentional and structural analysis dependent on musical hierarchies, which are enhanced in musicians.

## Introduction

The hierarchical organization of listeners’ perceptions of tensions and relaxations through time forms musical syntax [1]. Musical regularities (e.g. how musical events are combined into sequences) creates predictions about upcoming musical events or *schematic expectations* [2], by which unstable chords should lead to more stable ones. Our brain also makes predictions based on the probability of appearance of unexpected musical events, which generate *veridical expectations* (e.g., through the repetition of violations; [3,4]). One of the key questions of music cognition research is how the creation and violation of these musical expectations are encoded in the brain. In other words, how expectation and prediction interact with occurrence, as music perception unfolds over time. To address these issues, a large body of empirical research [5–8] studied the set of neural responses that are elicited by unexpected musical events. However, the music-specificity and the contribution of cognitive/sensory processes behind these responses are still under debate [9–11]. In the present study, we contribute to this line of research by exploring whether the processing of tonal expectations broken by strong dissonance is modulated by schematic and veridical expectations and musical expertise.

In musical terms, irregular chords that violate schematic expectations (for instance, by disrupting the expectation for resolution after a dominant chord) elicit an *early right anterior negativity* (or ERAN, which is a type of MMN), the P3 and the N5 [5,9,12]. Traditionally, the ERAN is suggested to represent the cognitive processing of a violation of music-syntactic rules. In more general terms, the brain makes predictions about upcoming events and aims to reduce the uncertainty of these predictions by comparing prior models to sensory inputs (which is known as Bayesian surprise; [13–15]). Unpredictable or surprising events are informative because they contribute to reduce the uncertainty of the brain’s predictions and, therefore, elicit neural responses, such as the MMN [16,17]). Interestingly, when irregularities become predictably surprising through repetition, the subsequent neural responses (such as the ERAN) diminish, as listeners extract veridical expectations about their appearance and become able to anticipate them [5,18]. It thus seems that, in music, schematic expectations are modulated to some extent by veridical expectations [3,19]. Likely, the reduction of the neural responses after exposure to unexpected events is related to the sensory cortex having evolved to stop responding to those events that match its predictions and are, therefore, uninformative [20].

Syntactic processes in music are intimately entwined with sensory-driven processes (in contrast to language). Western tonal syntax is deeply rooted in the acoustic properties of sounds, their psychoacoustic effects and their storage in auditory memory. For example, the syntactically most important events have also strong overlap in harmonic spectra, which is an acoustic feature. Thus, tonal hierarchies and their subsequent neural responses might be (at least partly) an emergent property of auditory short-term memory (ASTM), which is the overlap of the auditory image of any tone or chord with the auditory image created by the previous events accumulated in auditory memory [11,21].

A critical issue in music cognition is to determine the respective weights that acoustic information stored in ASTM and learned syntactic representations have in musical syntax processing [21]. To address that issue, most research focused on minimizing acoustic deviance and introducing only musical deviance [9,10]. Some studies explored chords that introduce pure acoustical deviance independently of their relationship with the musical context. For example, auditory deviants like mistuned chords [7] and frequency violations [22]. Koelsch and colleagues [5] compared the ERPs triggered by dissonant and syntactic violations of tonal context. In that study, highly dissonant semitone clusters introduced acoustic deviance without being syntactically correct. At the same time, Neapolitan chords introduced contextual acoustic dissimilarity (with their out-of-key tones) and were rendered either syntactically correct or incorrect depending on their position within the cadence. The critical finding of that study (that was later supported by other studies [7,9] was that the amplitude of the ERAN was larger for Neapolitans presented at the end of the cadence than for Neapolitans at the third position. At the third position, Neapolitan chords substituted a subdominant (*syntactically correct)* while at the end of the cadence sabotaged the resolution to the tonic (*syntactically incorrect*). The authors thus suggested that the ERAN reflects syntactic-like processes because irregular chords violate sound expectancy to a higher degree at the end of a cadence [5,7]. However, the authors also found a difference between positions for dissonant clusters, that were not syntactically correct at any of the positions, as if it was difficult for the brain to distinguish syntactic from acoustic violations. Moreover, more recent research demonstrated that ASTM can also account for the responses reported in most electrophysiological studies [11]. Thus, there is a current debate around the sensory and cognitive mechanisms behind music processing.

In the present study, we advance this line of research by using dissonant chords that introduced a strong acoustical violation but that, in terms of ASTM, [11,21], were undistinguishable between positions. Dissonant chords include intervals with complex frequency ratios, roughness, which creates the overlapping of amplitude envelopes in the basilar membrane [23] and a lack of harmonicity [24]. The convention of consonance/dissonance is an always-changing continuum [25]. For instance, the perception of seventh chords changed from dissonant in the 18th century to commonplace in classical music [26] or mildly dissonant and even preferred in jazz tradition [27,28]. In the present study we thus use chords deliberately containing highly dissonant intervals (such as the minor second and the tritone) that are very rare in most western genres [25,29] and do not act as culturally specific chord prototypes [30]. Thus, their interpretation should depend less on the musical experience of the listener while they represent a good model for investigating the role of prolonged experience on the ability to distinguish sensory from syntactic violations.

Musical training enhances the neural responses triggered by most musical violations. Western musicians show a larger responses to irregular chords because their expectations for tonal resolutions are more finely tuned [12]. Musicians are also more likely exposed to intentionally dissonant or mistuned chords, which are more frequent in genres like avant-garde or free jazz and contemporary music [27]. Thus, they display a flexible auditory system with a greater ability to quickly categorize dissonant chords [28,30–33] and more rapid perceptual learning [34]. The comparison of highly trained musicians with naïve listeners might provide valuable information about the discrimination between syntactic and acoustic violations.

Thus, in the present study, we investigate whether the neural responses to dissonance change as a function of the position that the chords occupy in a sequence (Experiment 1). Second, we study whether these responses are modulated by the predictability of dissonant endings (Experiment 2). To account for a possible role of training in how these conditions modulate the neural responses of the listener, in both experiments we compared highly trained musicians against naïve listeners.

## Experiment 1

### Introduction

In previous studies, syntactic irregularities (in the absence of acoustic deviance) were found to elicit stronger ERAN as closure than at middle positions of chord cadences [9,10], because at the end of the cadence they violate sound expectancy to a higher degree. The first chords of a chord sequence do not clearly establish a key (e.g., a C followed by Am can lead to 6 different possible keys). But the key is unequivocally established after four chords. Moreover, unstable chords (as the dominant at the penultimate position) increase the demand of resolution [35]. In terms of Bayesian surprise, precision estimates accumulate over time based on the probability distribution of each event [36]. Therefore, the auditory system collects more evidence in favour of in-key chords after 4 chords than after 2 chords.

In our first experiment, we investigated the neural responses elicited by acoustic violations in a tonal context, introduced by dissonant chords at different positions of a chord cadence. In contrast with previous research, we studied whether these responses are modulated by musical expertise. We presented trained musicians and naïve listeners with dissonant clusters placed at the third (middle) and at the fifth (ending) position of a chord cadence. Besides being highly dissonant, they both introduced an out-of-key tone but were indistinguishable between positions at the auditory level. Therefore, if we observe stronger responses at the ending position, they would be based on stronger tonality establishment and expectations of resolution (which, in turn, might be based on the accumulation of sensory evidence in favour of the tonic). On the contrary, if the responses to dissonance were not different between positions, it would suggest that dissonance is processed as an acoustic anomaly not affected by hierarchical relationships. We hypothesized that musicians might show a different pattern of neural responses than non-musicians.

## Methods

### Participants

24 volunteers participated in the experiment, most of them were undergrade students. 12 were musicians and 12, non-musicians [8,31,37]. Musicians (3 identified as cisgender women, mean age 23.66 ± 6.53), finished or were studying Advanced Studies in Western music, starting at 6.9 (± 2.2) years of age and had been musically active for 14.6 (±2.8) years on average. They all specialized in an instrument (mostly piano), 5 out of 12 studied jazz and modern music and all were musically active at the time of the experiment. Non-musicians (8 of which identified as cisgender women, mean age 24.3 ± 5.4) never received formal musical training besides the compulsory program at school. All participants were right-handed and reported normal (or corrected to normal) vision and no diagnosed hearing problems. The study protocol was approved by the Etical Committee for Drugs Research Parc de Salut Mar (Comité de Ética de la Investigación con Medicamientos Parc de Salut Mar, CEIm, under the reference number 2018/7888/I). We obtained written informed consent from the participants who received a monetary compensation for their participation of 5€ each 30min. The data was analyzed anonymously.

### Stimuli

**Fig 1:**
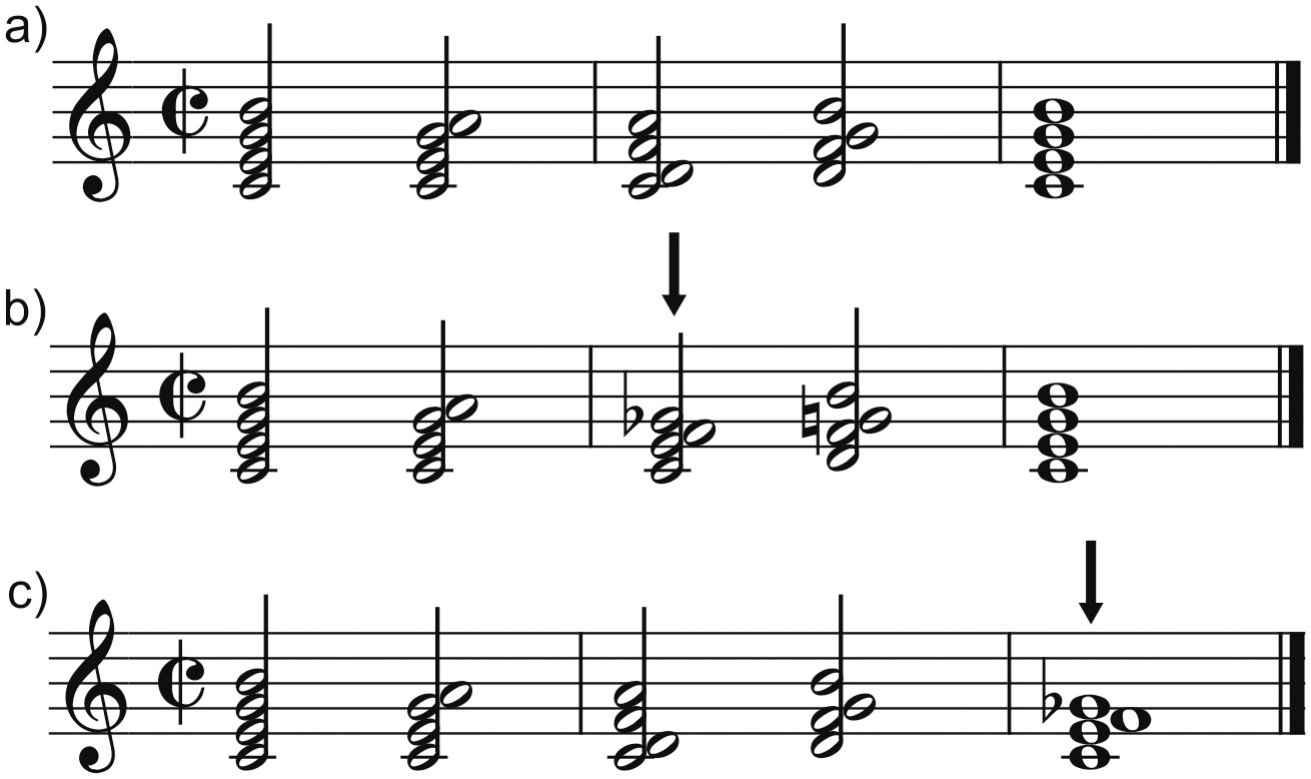
Examples of chord sequences in root position. Dissonant clusters are indicated by black arrows. a) Cadences consisting of in-key chords that resolve to the tonic. b) Cadences containing a dissonant cluster in the third position. c) Cadences containing a dissonant cluster in the fifth position.

We created different 5-chord sequences. Each chord was composed of four notes (with respect to the tonic: fundamental, third, fifth and seventh). The chord at the first position in each sequence was always the tonic seventh chord. The second chord could be the submediant (VIm7) or the subdominant (IVMaj7). The third chord could be the supertonic (IIm7), the subdominant (IVMaj7) or a dissonant cluster. The fourth chord was always the dominant seventh (V7) to create the maximum expectation for resolution. These combinations established three types of context sequences: I-VIm-IV-V, I-IV-IIm-V and I-IV-IIm-V. The sequences could end in either the tonic or a dissonant cluster (Fig 1). Dissonant chords kept the fundamental and the third of the tonic but included a cluster of semitones (with respect to the tonic: the third, the fourth and the augmented fourth). Thus, they were a dissonant version of the tonic chord, that is syntactically correct at both the third and the fifth position. Preliminary analyses with the ASTM model proposed by Leman [11,21] were performed to test the goodness of fit of dissonant clusters with the musical context. The ASTM model calculates the acoustic congruency of a chord with the auditory sensory memory traces established by the previous chords, whose auditory information decays but is kept in the echoic memory for a certain time. Thus, it calculates the correlation between the pitch image of a chord and the pitch image of the previous chords stored in the echoic memory [10]. According to the model, clusters in our study were acoustically more similar to the tonic than a Neapolitan violation, despite being highly dissonant, because they shared more tones with the tonic. More importantly, they were acoustically undistinguishable between positions. Therefore, any differences in the ERPs between positions could not be explained by differences in auditory deviance.

In order to add more variability to the stimuli, the three basic context sequences were modified so that the first and last chord were either in root position, first inversion or second inversion. The lead voice always moved by conjunct melodic motion. The resulting sequences were moreover transposed to the 12 different keys, resulting in 108 context sequences (without taking into account the sequences that had the dissonant ending at the third position). We manipulated the proportion of appearance of each type of target chord. Fifty percent of the total sequences (216) ended as expected (in the tonic). 108 sequences presented a dissonant cluster at the third position, and another 108 sequences presented a dissonant cluster at the fifth position (Fig 1). Thus, both kinds of sequences containing dissonant clusters appeared with a probability of 25% each. There was a total of 432 sequences played. Within each sequence, chords 1 to 4 lasted for 600ms each [5,12] and chord 5 lasted for 1200ms. Each sequence lasted for 3600ms. All sequences were recorded with Cubase LE AI Elements 9.5 using the piano instrument. In order to create a secondary task (see procedure below), 10% of the sequences (four randomly selected sequences of each tonality) included a chord in the second or third position played by a deviant instrument other than a piano (violin, guitar or vibraphone). When the sequence had a dissonant chord at the third position, it was never played by a deviant instrument. However, deviant instruments at the second position could be followed by a dissonant chord at the third position. The 36 sequences belonging to the same key were presented together in a block. Thus, there were 12 blocks (one per key). The order of presentation of blocks was randomized across participants.

### Procedure

The experiment was run in an acoustically and electrically shielded room. Participants sat comfortably in an armchair and listened to the sequences, presented via headphones using Matlab. Participants were told not to blink, move their body or their eyes while listening. They were instructed to look at a fixation cross at the centre of the screen. We presented the sequences with a silent period of 500ms between them, during which three stars appeared on the screen. That break between sequences performed a double function: first, it allowed participants to blink between sequences, which prevented having artefacts overwritten in the signal of interest. Second, a pause prevented that the responses to the last and longer chord overlapped with those elicited by the first chord of the next sequence. In order to keep the participants engaged while listening to the sequences, we used a secondary task. The participants were asked to detect and count how many times they heard an instrument different than a piano. They were not informed about the presence of dissonant ending chords to avoid overlapping attentional and decisional effects in the responses of interest. Twice in each block (every 18 sequences) the participants were asked by a text shown on the screen to provide a numerical answer with the keypad. At the end of each block there was a 10s countdown as a break. There was also a longer break in the middle of the experiment so participants could move, after which they had to press the SPACE bar to continue with the experiment. The duration of the entire experimental session was approximately 40 min.

### Recording and analyses

EEG measurements were recorded with a 32 channels actiCAP Slim (Brain Products) with Ag/AgCl electrodes. 28 scalp locations following the 10–20 system were recorded (Fp1, Fp2, F7, F3, Fz, F4, F8, FC5, FC1, FC2, FC6, T7, C3, Cz, C4, T8, TP9, CP5, CP1, CP2, CP6, TP10, P7, P3, Pz, P4, P8, Oz). Two electrodes were placed in the left and right mastoid (MSDL, MSDR) and two more in the outer side (HEOG) and below (VEOG) the right eye to monitor the ocular movements and blinking. Measurements were referenced online to the tip of the nose, and offline to the average of the mastoids. The sampling rate was 500Hz and impedances were kept below 10kΩ. Data was recorded using BrainVision Recorder and the triggers sent simultaneously with Matlab 2019b.

Data was pre-processed offline with Fieldtrip Toolbox: filtered from 0.4 to 40Hz and bad electrodes corrected via neighbour interpolation. Independent Component Analysis (ICA) was applied to the whole trial epochs (3.6s) to identify the variance caused by eye movements, heart and muscular movement. The components reflecting the artefacts were manually removed individually on each participant. On average, 2% of trials were rejected from further analysis because of residual excessive noise. After artefact rejection, the data was divided in epochs of 600ms for chords at the third position in Experiment 1 and of 1200ms for chords at the ending position (nevertheless, the responses of interest were always within the first 600ms). We applied a baseline correction from 200ms to the onset of the chord performed in two steps: first trial by trial and, after calculating average ERPs, a condition-specific baseline for all participants. Data for statistical analyses were selected from the time windows in which the responses of interest are usually found in the literature: 150-250ms for the early negativity, 300-450ms for the frontal positivity and 500-600ms for the late negativity [5,12,22]. We later confirmed these time windows by visually inspecting the grand-average difference waves (dissonant minus in-key chords). That visual inspection also revealed a parietal subcomponent of a positivity that was further analysed within the time window 350-550ms. Note that we were not interested in the effect of Chord itself, but rather in the interactions. To avoid priming effects on dissonant ending chords, we excluded from the analyses regarding the effect of dissonant chords those sequences containing deviant instruments. We moreover verified that the statistical results were not affected by the exclusion of these trials.

We calculated the ERPs by averaging across the trials belonging to each condition (in-key 3rd position: 324 trials, in-key 5th position: 216, dissonant 3rd position: 108, dissonant 5th position: 108). Then, for each time window of interest, we calculated the mean amplitudes for each participant in each condition. After, we averaged these mean amplitudes across the electrodes of our regions of interest (ROIs) (right-frontal electrodes [F4, F8, FC2, FC4], left-frontal electrodes [F3, F7, FC1, FC3]) based on previous literature regarding the responses of interest [5,12], which entered the statistical analysis. We performed mixed-design repeated measures ANOVA with the within-subject factors Region of Interest (ROI; left-anterior, right-anterior), Chord Type (in-key, dissonant), Position of the dissonant chord within the sequence (3rd, which corresponds to a lower expectation violation and 5th, a high expectation violation) and the between-subjects factor Group (musicians, non-musicians). To analyse the parietal response, parallel ANOVAs were run over the parietal ROIs (right-parietal [CP2, CP6, P4, P8], left-parietal [CP1, CP5, P3, P6]). We also analysed the responses to deviant instruments, by comparing the amplitudes of the windows 150–200 and 250-400ms with the factors Instrument (piano vs deviant), ROI and Group. In all the analyses, to correct for multiple comparisons (in all the tests over different time windows), we applied a Bonferroni correction and when sphericity was violated, results with Greenhouse-Geiser correction are reported.

## Results

### Dissonant chords

**Fig 2:**
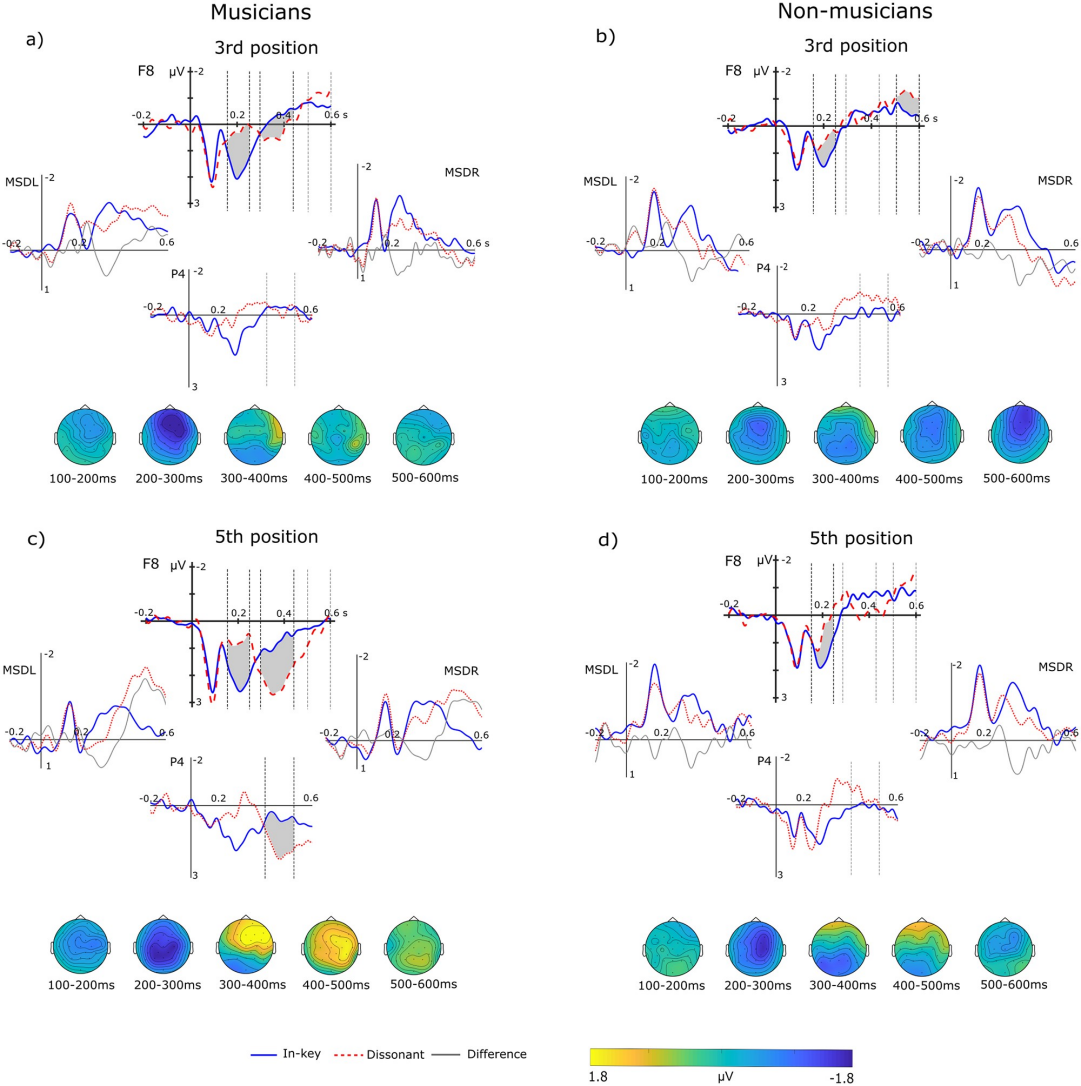
Experiment 1: Event-related potentials of musicians and non-musicians. Graphs include grand-average ERPs and the topographical distribution of the difference waves (dissonant minus in-key) of musicians (a, c) and non-musicians (b, d) at the third (a, b) and fifth position (c, d). Dotted vertical lines indicate the limits of the time windows of interest analysed. Shaded areas correspond to significant ERP effects, which are best seen in electrode F8. Left (MSDL) and right mastoidal (MSDR), and parietal (P4) are also shown. Topographical plots depict the grand-average difference wave to better observe the evolution of the topographical distribution of each ERP of interest.

We observed a negative response for dissonant chords in comparison to in-key chords in both the third and the fifth positions around 200ms with a right-frontal distribution (Fig 2). We also observed a frontal positivity peaking around 350ms and a late parietal positivity arising around 400ms (Fig 2). Finally, we observed a bilateral late negativity arising around 500ms.

For the 150-250ms time window, the ANOVA yielded a main effect of Chord (F(1, 22) = 42.46, p < .001, η2 = .66), ROI (F(1, 22) = 6.50, p = .018, η2 = .23) and Position (F(1, 22) = 8.90, p = .007, η2 = .29). It also showed interactions between Chord and Group (F(1, 22) = 5.32, p = .031, η2 = .20), Chord and ROI (F(1, 22) = 7.06, p = .014, η2 = .24) and Position and ROI (F(1, 22) = 8.86, p = .007, η2 = .29). The effect of Chord suggested that dissonant chords elicit a negativity in comparison to in-key chords in both groups (mean amplitude difference between chord types ± SEM: -1,176 ± 0.18). The amplitude of the negativity appeared larger in musicians (-1.59 ± 0.26) than in non-musicians (-0.76 ± 0.26) and in the right ROI (-1.30 ± 0.21) than in the left ROI (-1.05 ± 0.21), which could suggest a right-lateralization of the response. Importantly, the lack of interaction between Chord and Position indicated that the magnitude of the effect did not depend on whether the dissonant chord was presented in the middle of the sequence (third position) or at its end (fifth position; Table 1).

**Table 1:** Mean amplitudes and SEM for the ERPs of interest. Mean amplitudes reported are the grand-average of the mean amplitude of the difference waves calculated within each time window of interest.

			Mean amplitude (μV) + SEM			
			EN	P3a	P3b	N5
Experiment 1	Musicians	3rd position	-1.65 (0.22)	0.21 (0.22)	0.08(0.22)	-0.17 (0.21)
		5th position	-1.52 (0.39)	1.56 (0.42)	0.96(0.30)	0.42 (0.36)
	Non-musicians	3rd position	-0.71 (0.30)	-0.34 (0.28)	-0.6(0.19)	-1.01 (0.24)
		5th position	-0.83 (0.23)	0.36 (0.33)	-0.27(0.18)	-0.37 (0.18)
Experiment 2	Musicians		-1.20 (0.33)	1.90 (0.56)	0.90(0.28)	0.25 (0.27)
	Non-musicians		-1.34 (0.34)	0.73 (0.33)	0.37(0.35)	-0.98 (0.23)

For the 300-450ms time window, the ANOVA yielded a main effect of Chord (F(1, 22) = 6.24, p = .021, η2 = .22), Position (F(1, 22) = 23.74, p < .001, η2 = .51) and Group (F(1, 22) = 20.82, p < .001, η2 = .49). Also, it revealed interactions between Chord and Position (F(1, 22) = 12.81, p = .002, η2 = .37), Chord and Group (F(1, 22) = 6.01, p = .023, η2 = .21), Chord and ROI (F(1, 22) = 8.02 p = .010, η2 = .27), ROI and Group (F(1, 22) = 18.40, p < .001, η2 = .46), Position and Group (F(1, 22) = 8.84, p = .007, η2 = .29), ROI, Chord and Group (F(1, 22) = 5.11, p = .034, η2 = .19) and between Chord, Position, ROI and Group (F(1, 22) = 5.10 p = .034, η2 = .19). Together, these effects suggested that dissonant chords elicit a positivity in comparison to in-key chords in this time window (mean difference 0.45 ± 0.18). Post hoc tests for the interactions revealed that this effect reached statistical significance for chords at the 5th position in musicians (0.89 ± 0.26, p < .001, 95% CI = 1.11, 2.65) but not in non-musicians (0.01 ± 0.26) and was larger in the right ROI (1.89 ± 0.37 vs 1.25 ± 0.41).

For the 350-550ms time window, the ANOVA yielded a main effect of Position (F(1, 22) = 71.45, p < .001, η2 = .77), ROI (F(1, 22) = 9.54 p = .005, η2 = .30), Group (F(1, 22) = 13.64, p = .001, η2 = .38) and interactions between Chord and Group (F(1, 22) = 18.54, p < .001, η2 = .46), Position and Group (F(1, 22) = 22.81, p < .001, η2 = .51) and Position and Chord (F(1, 22) = 6.40, p = .019, η2 = .23). Post hoc tests for interactions suggested that dissonant chords elicited a positivity in comparison to in-key chords, which was larger in musicians (0.52 ± 0.16) than in non-musicians (-0.43 ± 0.16), as the effect of Chord only reached significance in the former group (p < .003, 95% CI = 0.20, 0.84). The positivity also appeared larger at the fifth than at the third position (0.35 ± 0.18 vs -0.25 ± 0.15).

For the 500-600ms time window, the ANOVA revealed a main effect of Chord (F(1, 22) = 4.99, p = .036, η2 = .19), Group (F(1, 22) = 5.34, p = .031, η2 = .20) and Position (F(1, 22) = 12.46, p = .002, η2 = .36). Moreover, there were interactions between Chord and Group (F(1,22) = 9.79, p = .005, η2 = .31), Chord and Position (F(1, 22) = 5.52, p = .028, η2 = .20), ROI and Group (F(1, 22) = 8.13, p = .009, η2 = .27) and between Position, ROI and Group (F(1, 22) = 4.58, p = .044, η2 = .17). The effect of Chord indicated that dissonant chords elicit a negativity in comparison to in-key chords (-0.31 ± 0.14). Post hoc tests for interactions suggested that the Chord effect was significant only at the third position (p = .001, 95% CI = -0.92, -0.26) and was larger in non-musicians (-0.74 ± 0.20) than in musicians (-0.12 ± 0.20) as it only reached significance in the former group (p = .001, 95%CI = -1.15, -0.34).

### Deviant instruments

**Fig 3:**
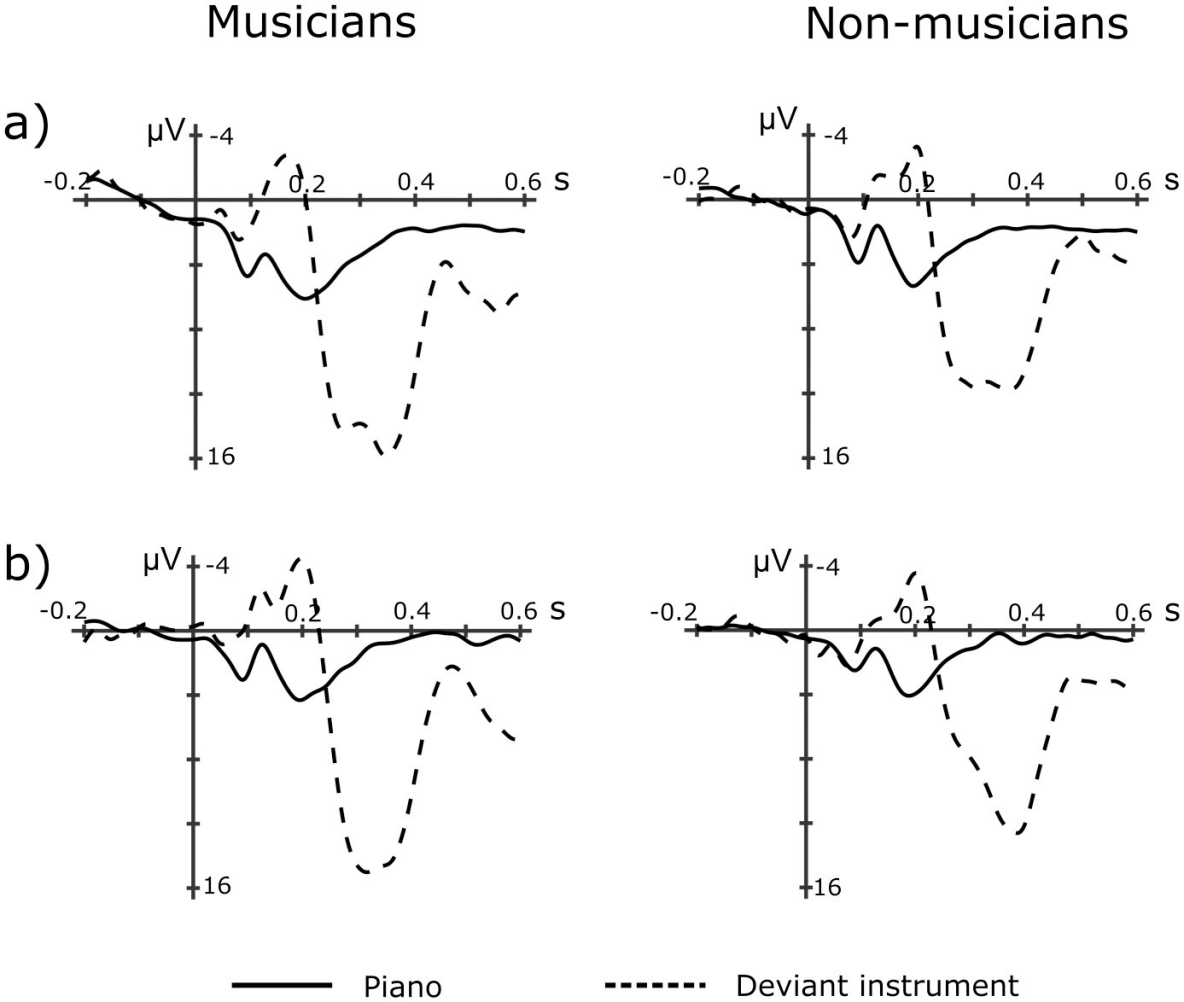
Event-related potentials of deviant instruments. Grand-average ERPs for deviant and standard (piano) instruments for musicians and non-musicians for Experiment 1 (a) and Experiment 2 (b). Potentials at Cz are being depicted.

The presentation of deviant instruments elicited a central negativity that peaked around 180ms. The negativity was followed by a large frontal positivity peaking around 300ms and a late parietal positivity arising around 450ms (Fig 3).

For the 150-200ms time window, we found an effect of Instrument (F(1, 22) = 28.47, p < .001, η2 = .56, 95% CI = -5.48, -2.41). The magnitude of the negativity did not differ between groups. For the 250-400ms time window we found a main effect of Instrument (F(1, 22) = 109.30, p < .001, η2 = .83, 95% CI = 5.42, 8.10) and of Group (F(1, 22) = 10.88, p = .003, η2 = .33, 95% CI = 0.85, 3.71). As suggested by the lack of interactions with Group, deviant instruments elicited comparable responses between musicians and non-musicians.

In the behavioural task of counting the number of deviant instruments, we calculated the performance of participants as the number of correct counts of deviant instruments over the total of times that they were asked. Musicians had a performance of 92.36% ± 2.80 and non-musicians, of 88.89% ± 2.96. A good performance in both groups of participants indicated that they paid attention to the task. Moreover, the performance was not perfect in either group, indicating that the task was not too easy, not even for musicians.

## Discussion

In the present experiment dissonant clusters elicited a right-frontal early negativity (which we will refer to as EN for simplicity) that reached its maximum around 200ms. The amplitude of the EN did not significantly differ between positions, but it was larger in musicians than in non-musicians. Moreover, we found a robust frontal positivity peaking around 300ms (taken as the P3a) that was larger in musicians for ending clusters and was followed by a late parietal positivity (taken as the P3b). Finally, clusters elicited a late bilateral negativity at 500ms (taken as the N5), which was most prominent in non-musicians for middle clusters.

### Early negativity

Dissonant clusters elicited an EN in musicians and non-musicians. The amplitude of the EN was statistically undistinguishable between the middle and the ending positions in both groups. Syntactic irregularities well controlled for acoustic deviance have been shown to trigger larger negativities for ending positions [9] that cannot be simply accounted by ASTM [11]. In our experiment, clusters did not differ across positions in terms of ASTM. Because early responses are more related to basic acoustic deviations, the similarity in the EN across positions that we observed may reflect the detection of the acoustic deviance introduced by the distinction between consonant/dissonant (which did not differ across positions) and may be simply interpreted as a MMN [7,38]. However, such early response may not be sensible to higher-level processes related to schematic expectations or the degree of sensory surprise based on the evidence in favour of the tonic accumulated across the cadence. That is a striking result, especially in musicians, who are able to automatically categorize chords and should form stronger expectations.

Importantly, musicians showed a larger EN than non-musicians. Musicians have enhanced discrimination of dissonance at early time windows [30,39,40]. However, the contributions to this enhancement are under current debate. One possibility is that musicians possess more sophisticated overall auditory abilities for any kind of sound. This may provide them with greater sensitivity for the beats in the dissonant chord (as shown in the N2; [41]). However, an enhancement in sensory processing should also be reflected in the initial feature analysis in the auditory cortex, and signalled by the P1 or the N1, which is not the case [30]. A complementary view is that musical training provides listeners with a perceptual expertise that allows them to differentiate culture-specific musical categories, such as non-prototypical chords [30]. Indeed, musicians’ MMN is equally sensitive to non-prototypical dissonant chords in a dissonant context than to stereotypical chords in a consonant context [40,42]. Moreover, different types of musical training might separately shape sensory and categorization abilities. For instance, jazz training includes ear learning together with explicit knowledge of complex chord changes and rich harmonies, which can be reflected in a general enhancement of the MMN in comparison to classical or pop musicians [43]. Our results are consistent with both explanations and might emerge from the combination of both the sophistication of auditory abilities and the perceptual expertise that derive from musical training.

### P3 effects

Dissonant clusters elicited a frontal positivity around 300ms, which is consistent with the P3a (Fig 2) and a late parietal positivity, interpreted as the P3b. The P3a and P3b are considered subcomponents of the P300, where the P3a (novelty P3 in traditional terms) is frequently linked to the non-intentional allocation of attention resources to a stimulus that is novel or unexpected [44,45] and the P3b (or target P3) is associated with context updating (see below). In terms of sensory surprise, stimuli with a low likelihood given a distribution of expected or learned stimuli are more informative, more surprising [20]. Precisely because unexpected events are informative, they require further evaluation, for which is necessary the attention of the listener. If sensory changes were predictable, further evaluation would not be necessary [46]. Therefore, that task-relevant deviant instruments were surprising and attracted the attention of participants was expected because the participants were informed about their presence and instructed to detect them. But, importantly, participants were not informed about the presence of dissonant chords [5,12]. Therefore, that clusters elicited the P3a could suggest that dissonant chords were surprising, salient or informative enough for listeners and subsequently attract their attention, because we actively seek or attend to sensory cues that are surprising [47]. This is in line with the idea that, for the P3a to be elicited, it requires that the change exceeds a certain threshold to be salient [46].

**Fig 4:**
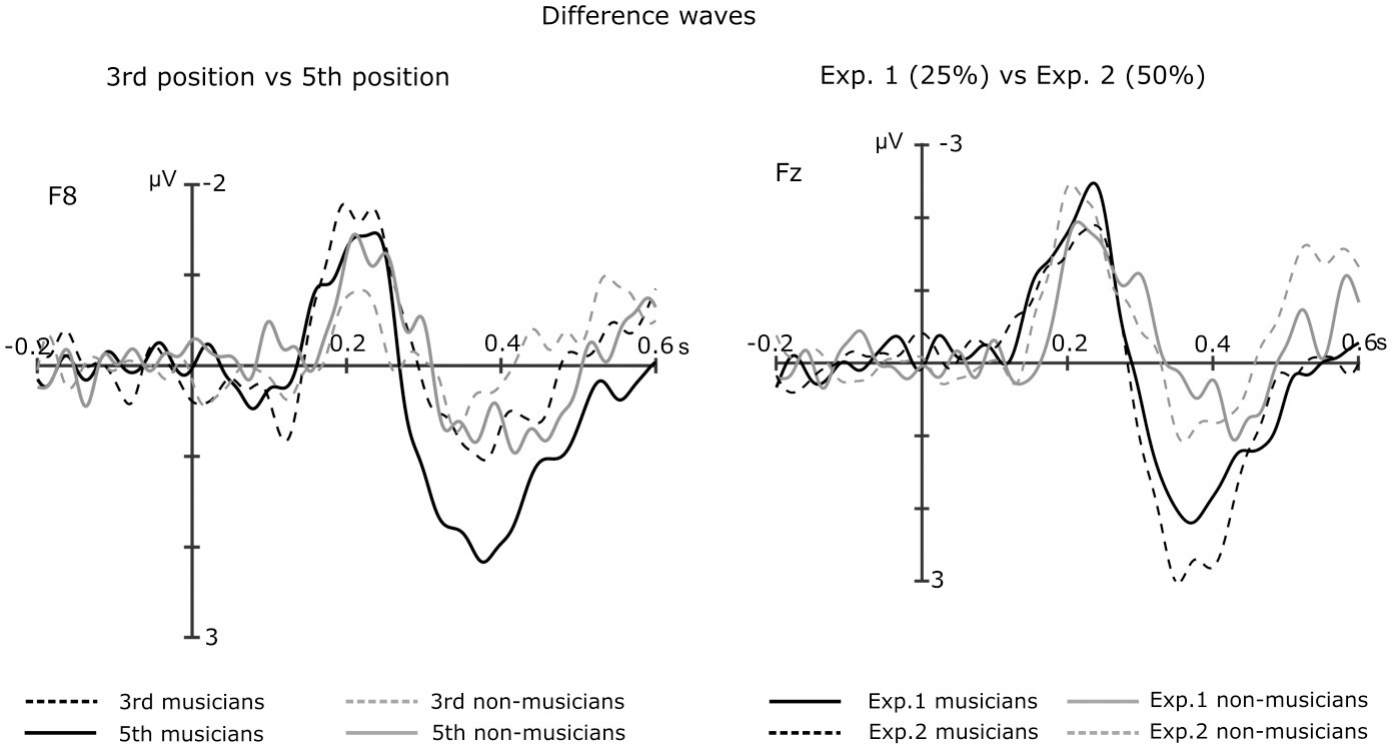
Difference waves the ERPs of musicians and non-musicians. Left panel: Difference wave comparing chords at the 3rd versus the 5th position. Right panel: Difference wave comparing Experiment 1 (where dissonant endings occurred with a probability of 25%) versus Experiment 2 (where dissonant endings occurred with a probability of 50%).

Importantly, the P3a elicited by dissonant clusters was statistically significant in musicians but not in non-musicians. Musical training is known to enhance P3 effects [2,48]: musicians have better perceptual learning, and stronger and faster involuntary attention switching [49]. Thus, the lack of a significant in the P3a in non-musicians might suggest that the change that clusters introduced in the chord sequences might have not been as surprising or salient for them than for musicians. Furthermore, in musicians, the P3a was significantly larger for ending than for middle clusters (Fig 4, left panel). Thus, strong dissonance at the end of a cadence might be perceived as less expected, more surprising, than in the middle position. In musical terms, ending clusters are more surprising than middle clusters because they substitute the very expected tonic, while at the third position they substitute a subdominant. Similarly, within a framework of Bayesian surprise, ending clusters are more surprising because the sensory system has accumulated more evidence in favour of the tonic by the fifth position.

Ending clusters, but not middle clusters, elicited a later parietal P3b that was significant in musicians but not in non-musicians. The P3b is related to subsequent memory processing arising from the revision of the brain’s current model of the musical structure induced by incoming stimuli [50] or, in other words, context-updating [51]. The P3b is also related to processes of structural analysis in rule-governed sequences [50], which reflects proper conscious perception [45] and ultimately leads to coordination of behavioural responses [5]. Importantly, the amplitude of the P3b reflects the amount of information extracted from the unexpected stimulus [46]. This suggests that ending clusters might have recruited further processing resources than middle clusters because they introduced a larger disruption of the current sensory/musical model. At the same time, the P3b is thought to be elicited only by task-relevant stimuli [46], suggesting that such disruption was surprising enough to overcome task relevancy. The fact that ending clusters elicited a P3b that was significant only in musicians suggests that they might be more efficient at matching the target chord to the memory trace of the context [49] than non-musicians [5]. Indeed, in our study some musicians reported that they were strongly surprised by dissonant chords and were tempted to respond to them.

In sum, in contrast to early responses, the subcomponents of the P3 elicited by dissonant chords suggest that ending clusters became more surprising than middle clusters, especially for experienced listeners. Importantly, acoustic differences in terms of ASTM cannot account for such enhancement of the P3 subcomponents toward the end of the cadence, suggesting that these late responses are sensible enough to register higher-order schematic expectations.

### N5 effects

Dissonant clusters elicited a negativity around 500ms consistent with the N5. However, the N5 was statistically significant for middle clusters in non-musicians, but not in musicians. The N5 has been reported when irregular chords are task-irrelevant [12,52]. That we observed the N5 at least in non-musicians suggests that they made an effort in integrating clusters into the previous context [5]. On the other hand, it is unlikely that the lack of a N5 in musicians is due to an ease in the integration of clusters, because clusters are very strong acoustic violations and musicians are very sensitive to dissonance [30,32,53]. A possible explanation is that the positive potentials of the large P3 effects compensated the negative potentials of the N5 [5,9]. This reduction effect would be most salient in musicians, who showed a clear P3a. Similar effects have been observed when participants were asked to respond to irregular chords, which increased the P3 amplitude for target deviants [5,12]. Similarly, the N5 that we observed in non-musicians was larger for middle than for ending clusters, likely because the increasing magnitude of the P3a toward the end of the cadence increasingly masked the N5. The fact that the P3a possibly masked the N5 leaves open the question of whether musicians did not invest neural resources in integrating clusters because they are so unacceptable that are processed independently of music rules (while non-musicians attempted to do so) or whether musicians did but that was not reflected in the N5 due to the overlapping attentional effects.

Thus, in Experiment 1, we investigated whether the processing of dissonance is modulated by the syntactic relations in a chord cadence. We inserted infrequent dissonant chords in either the third or the fifth position of the cadence (each in 25% of the sequences). This design might tap into the interaction between veridical and schematic expectations because the appearance of a dissonant chord in the third position predicts that a dissonant chord will not appear in the ending position. To specifically test whether listeners can extract online higher-order veridical expectations about the appearance of dissonant closures, in experiment 2 we manipulated the predictability of dissonant closures by increasing their frequency of appearance.

## Experiment 2

### Introduction

The neural correlates responsive to irregularities (such as the MMN/ERAN and P3) have been suggested to be reduced by veridical expectations [5,18,51]. The precision of the brain’s predictions is contextual, because they accumulate over time, based on the statistics of the environment (e.g., the stimuli during an experiment). Listeners can accumulate evidence for the relative precision about both the local model (consonant/dissonant) and about the regularity of the pattern of stimuli itself (highly frequent dissonant chords), creating superordinate expectations. After exposure to a very predictable context for enough time, higher-order predictions are held with greater confidence, making them relatively impervious to disconfirmatory sensory evidence (which minimizes the subsequent responses) [36]. In other words, as initially unexpected events become predictable with repeated exposure, neural responses attenuate because they become uninformative for the brain’s internal models [20].

In the present experiment, we tested whether an increased predictability of acoustic violations in a tonal context (introduced by dissonant endings) would elicit reduced neural responses. We exposed listeners to musical sequences that, with a high probability (50%), would end on a dissonant cluster instead of the expected tonic. In this setting, the representation of correctly ending sequences would be weaker and clusters should become easier to anticipate, in comparison to Experiment 1, where clusters equally appeared at the middle or ending position. However, given the evidence suggesting that dissonance is psychoacoustically hard to process [53] and disruptive at the auditory level, we hypothesized that the neural responses to dissonant clusters would hardly diminish, even if listeners can easily anticipate their appearance. Alternatively, if the neural responses to frequent dissonant endings are in fact reduced in comparison to infrequent dissonant endings, it would suggest that veridical observations about any acoustical irregularity that appears often enough could prevail over the learned rules of hierarchical expectations, as if the irregularity becomes the new rule. Even music with quartertones sounds unpleasant at first, but after some repetitions, listeners tend to like it more as a result of the “mere exposure” effect [26]. Importantly, long-term musical training might lead to an increased experience with dissonant chords, faster perceptual learning and an ease in creating higher-order predictions for musical stimuli. Thus, we sought to explore the role of musical training in processing highly predictable dissonant closures. Musicians may render the anticipation of clusters easy enough to (at least partly) compensate the strong acoustic disruption that they introduce.

## Methods

### Participants

The participation criteria were identical to those in Experiment 1. 12 musicians (5 of which identified as cisgender women, mean age = 23 ± 4.3 years) and 12 non-musicians (3 identified as cisgender women, mean age = 23 ± 3.3 years) were recruited, none of whom had taken part in Experiment 1. Musicians started playing their instrument at a mean age of 8.1 ± 3.3 and had been playing for 14.1 ± 2.9 years on average. 5 out of 12 musicians studied modern music/jazz.

### Stimuli

The stimuli were identical to those used in Experiment 1 except for the proportion of appearance of chords. In the present experiment, 50% of the sequences ended with the tonic and 50% ended in a dissonant cluster. Contrary to Experiment 1, there were no dissonant clusters at the third position of the sequence.

### Procedure

The experimental procedure, including the secondary task, was identical to that of Experiment 1. Each experiment was ran separately.

### Analyses

To compare the mean amplitudes of the ERPs between conditions, we performed mixed-design repeated measures ANOVA with the within-subject factors Region of Interest (ROI; left-anterior, right-anterior), Chord Type (in-key, dissonant) and the between-subject factor Group (musician, non-musician). Moreover, for assessing the effect of proportion of clusters as ending chords, we compared the mean amplitudes of Experiment 1 (where 25% of sequences ended in a dissonant cluster) and Experiment 2 (where 50% did) with a between subjects univariate ANOVA with the amplitudes of the difference waves and the fixed factors Experiment (25% vs 50%) x Group (musicians vs non-musicians).

## Results

### Dissonant chords

**Fig 5:**
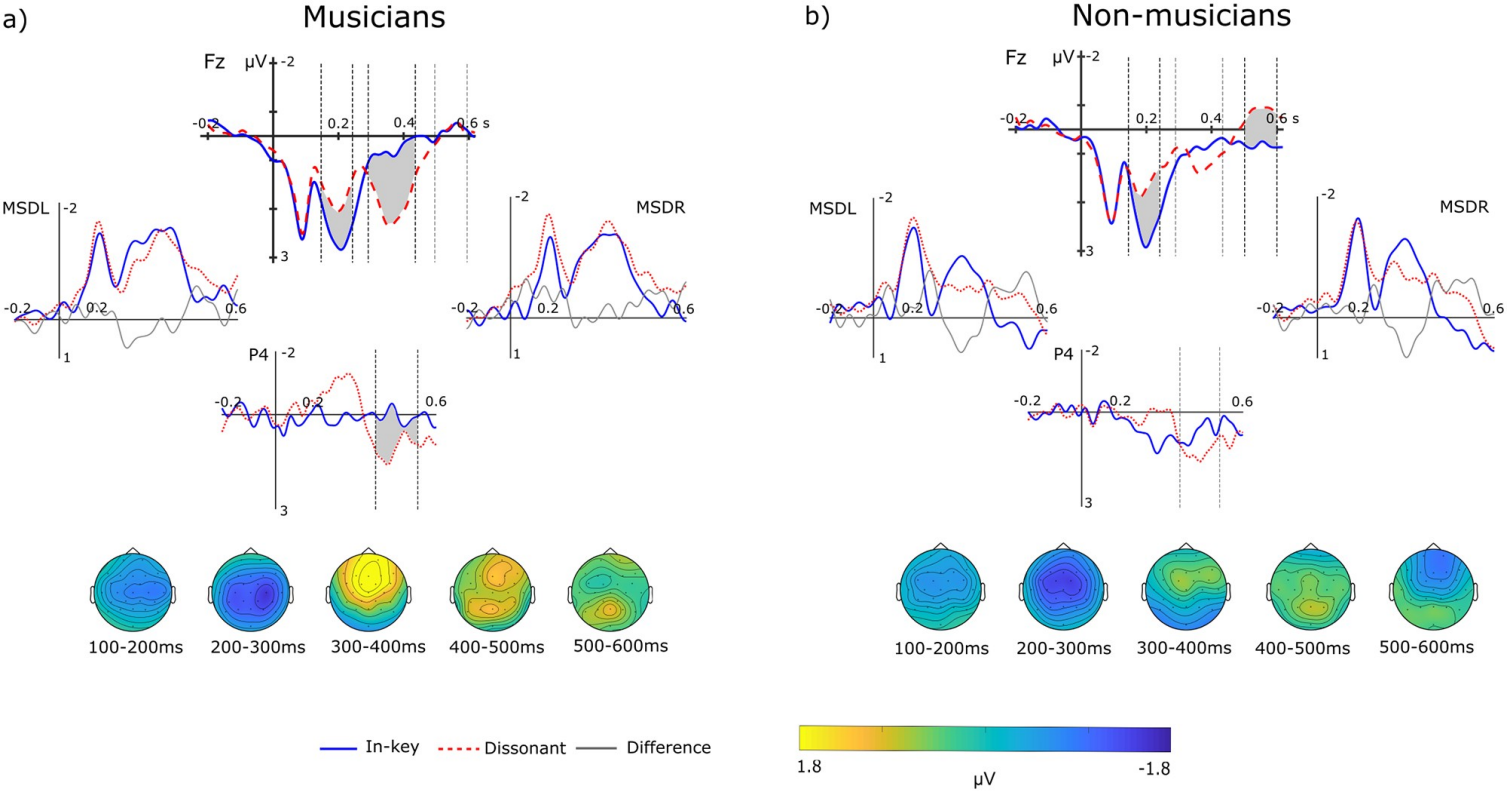
Experiment 2: Event-related potentials of musicians and non-musicians. Graphs include grand-average ERPs and topographical distribution of the difference waves (dissonant minus in-key) of musicians (a) and non-musicians (b) appearing in 50% of the sequences. Dotted vertical lines indicate the limits of the time windows of interest analysed. Shaded areas correspond to significant ERP effects, which are best seen in electrode F8. Left (MSDL) and right mastoidal (MSDR), and parietal (P4) are also shown. Topographical plots depict the grand-average difference wave to better observe the evolution of the topographical distribution of each ERP of interest.

We observed a negative response for dissonant chords in comparison to in-key chords peaking around 200ms with a fronto-central distribution. We also observed a frontal positivity around 350ms followed by a late parietal positivity and a late frontal-bilateral negativity that was only evident in non-musicians (Fig 5).

For the 150-250ms time window, the ANOVA revealed an effect of Chord (F(1, 22) = 29.23, p < .001, η2 = .57, 95% CI = -1.76, -0.78) which suggested that frequent dissonant chords in comparison to in-key chords elicit a negative response in both groups (-1,27 ± 0.24). Such effect was not lateralized, as indicated by the lack of interaction between Chord and ROI. Moreover, the lack of interaction between Chord and Group indicated that there was no difference between musicians and non-musicians. This contrasted with the responses that we observed in Experiment 1, where there was an enhancement in the EN in musicians.

For the 300-450ms time window, the ANOVA revealed an effect of Chord (F(1, 22) = 16.44, p = .001, η2 = .43, 95% CI = 0.53, 1.68) as dissonant endings elicited a positivity in comparison to in-key chords (1.31 ± 0.32). The interaction between Chord and Group (F(1, 22) = 3.23, p = .085, η2 = .13) was only marginally significant, with musicians having a slightly larger response than non-musicians (1.90 ± 0.46 vs 0.73 ± 0.46), as supported by the post hoc test (p < .001, 95% CI = 0.948, 2.850 in musicians).

For the 350-550ms time window the ANOVA yielded an effect of Chord F(1, 22) = 8.02, p = .010, η2 = .27), suggesting that dissonant chords elicited a P3b.

For the 500-600ms time window, the ANOVA yielded a main effect of Chord (F(1, 22) = 4.36, p = .049, η2 = .17) and an interaction between Chord and Group (F(1, 22) = 12.04, p = .002, η2 = .35). Thus, dissonant endings elicited a negativity in comparison to in-key chords (-0.37 ± 0.18). The negativity was larger in non-musicians than in musicians (0.98 ± 0.25 vs 0.24 ± 0.25), as supported by the post hoc test, that reached significance in non-musicians (p = .001, 95% CI = -1.50, -0.46).

#### Deviant instruments

Deviant instruments elicited a central negativity peaking around 200ms and a fronto-central positivity around 300ms (Fig 3). The ANOVA confirmed these responses for both the 150-200ms time window (F(1, 22) = 45.12, p < .001, η2 = .67, 95% CI = -4.62, -2.44) and the 300-400ms window (F(1, 22) = 212.01, p < .001, η2 = .91, 95% CI = 6.87, -9.15). We observed no difference between groups.

The performance in the behavioural task was 86.11% ± 5.55 in musicians and in non-musicians, 85.41% ± 5.34. As in Experiment 1, this suggests that participants were paying attention to the task in a consistent manner during the whole experiment.

#### Effect of proportion (Experiment 1—25% vs Experiment 2—50%)

Both in Experiment 1 and Experiment 2, clusters elicited similar responses at the time windows of interest (best seen in difference waves, Fig 4, right panel). To explore whether the amplitude of these ERPs was in fact affected by the predictability of ending clusters we compared them between experiments.

For the 150-250ms time window, the ANOVA showed that the main effects of Experiment and Group were non-significant.

For the time windows of the subcomponents of the P3b (300-450ms and 350-550ms), the effect of Experiment was non-significant. However, the main effect of Group was significant for both time windows (F(1,44) = 15,45, p < .001, 95%CI = 0.51, 1.60 and F(1, 44) = 7.821, p = .007, 95%CI = 0.33, 2.04, respectively), which could indicate that the P3a and P3b was larger in musicians in both experiments.

For the 500-600ms time window, the main effect of Experiment was non-significant but the effect of Group was significant (F(1,44) = 9.44, p = .004, 95%CI = -1.45, -0.30), which might indicate that the N5 was larger in non-musicians in both settings.

These results suggest that the amplitude of the ERPs of interest were not significantly modulated by the proportion of appearance of dissonant chords as cadence closures.

## Discussion

In the present experiment, dissonant clusters presented frequently elicited an EN with a fronto-central distribution in both musicians and non-musicians. Clusters also elicited a P3a and a P3b that were significant in musicians and an N5 that was significant in non-musicians. The amplitude of these responses did not significantly change when compared with those observed in Experiment 1.

### Early negativity

The comparison between Experiment 1 and Experiment 2 showed that the amplitude of the EN is independent of the predictability of dissonant chords as cadence closures, at least for the present manipulation.

The most likely explanation could be that the syntactic hierarchy of the cadences (or even the organization of chords into cadences) might not be accessible at early stages of processing clusters. That could be linked to the psychoacoustic difficulty in processing roughness. Clusters represented 10% of all the chords presented. Thus, the alternation of consonant and dissonant chords may have simply elicited a MMN as in an oddball paradigm. Moreover, in both experiments, the same number of sequences (50%) contained a cluster (either always at the end or in two positions), causing that the proportion of sequences containing *a* cluster was the same across experiments. However, previous studies with similar paradigms showed that the responses to syntactic irregularities (such as a Neapolitan sixth or a supertonic as a cadence closure) decrease after enough repetitions [5,18]. Thus, future research should address whether the lack of effect of predictability is due to the psychoacoustical difficulties for processing roughness [54] or whether is more related to a difficulty in extracting the experimental regularities.

### P3 effects

Frequent ending clusters elicited a P3a and a P3b, which were significant in musicians (Fig 5), but not in non-musicians. When compared with the results from Experiment 1, we observed that the amplitude of the P3 did not change as a function of the proportion of appearance of dissonant endings. That is in contrast with previous research, that shows that the P3a and P3b decline after the exposure to repeated syntactic irregularities [19,55], as listeners become able to predict the appearance of irregular events [56]. Moreover, musicians show habituation of the P3a during passive exposure to pitch deviants while non-musicians show an enhancement of that neural signature [49]. That the P3 did not decrease in our study after sustained repetition of clusters could be due to the fact that dissonant clusters are a very disruptive violation of sound expectancy based on the consonance dimension. Moreover, a dissonant closure of a chord cadence can hardly become less surprising, even if it was becoming easier to anticipate.

### N5 effects

Frequent dissonant clusters elicited a N5 that was significant in non-musicians. Previous studies argue that, when listeners familiarize to frequently occurring violations (like Neapolitans that can act as subdominants), their integration is facilitated [5]. However, clusters are very unlikely to be perceived as a function of the key, independently of how often they appear. Thus, the presence of the N5 suggests that frequently appearing ending clusters are still difficult to integrate in the cadence, at least for non-musicians. Similarly, to Experiment 1, the strong attentional effects (P3a) elicited by dissonant clusters might have masked the N5 in musicians.

## General discussion

In the present study, we investigated to what extent the neural responses to acoustic violations introduced by dissonant chords are modulated in a similar manner as the responses to syntactic irregularities are [5,9,10], with the aim to assess to what extent they share neural resources. The factors that we took into account were the musical training of the listener, and the musical and experimental context. First, we aimed to clarify whether the neural responses elicited by dissonant chords depend on the syntactic hierarchies created by chord cadences. Second, we investigated to what extent an increased predictability of dissonant closures of cadences modulates the subsequent neural responses. We found that unexpected dissonance elicited responses related to the detection of the unexpected event and subsequent responses related to the attraction of attention of the listener, structural analyses based on internal model updates and the integration into the previous context. We also found that musical training shapes how each of these neural responses differently interact with syntactic and experimental regularities.

The present study shows that both musicians and non-musicians readily detect dissonant clusters as an irregularity, as reflected by the EN that we observed in Experiments 1 and 2. The EN that we observed was not only a N2b because it inverted polarity at mastoid leads (Figs 2 and 5) which the N2b does not [9,57]. Even if more evidence is accumulated in favour of in-key chords and expectation for resolution are stronger at the end of the cadence, the amplitude of the EN did not significantly differ depending on the position of dissonant chords. This is in line with previous research showing that the acoustic deviance of dissonant chords is processed independently of their harmonic expectedness [32]. Moreover, even if the predictability of dissonance as a cadence closure was high, the amplitude of the EN did not decrease. Thus, early responses might be sensitive to the acoustic deviance introduced by dissonance (which did not differ across positions in terms of ASTM), but not to the higher-order schematic expectations (induced by syntactic regularities) and veridical expectations (induced by experimental regularities about frequency of appearance). Dissonance engages psychoacoustic constraints [54] because the roughness that it introduces is relevant at the auditory level, even to listeners with amusia [58,59]. Besides introducing an out-of-key pitch (*f#*; [9,10,21] clusters in the present study engaged a process of pure sensory analysis based on the roughness and beating of the chord [30,60]. Thus, the auditory system may have simply reacted to clusters as infrequent dissonant auditory deviants among frequent consonant chords [7,22]. By contrast, the early responses to syntactic deviance in the absence of acoustic deviance is in fact modulated by syntactic [9] and experimental regularities [18]. Here, we provide evidence that syntactic and acoustic deviance might engage different processes at early latencies. This pattern of results in music resembles language in the sense that syntax and changes in the acoustic information (phonetics) can be processed separately. Our results might be thus relevant to the current debate about whether music and language share neural resources [61,62 but see 11].

Previous studies had already reported increased MMN responses for dissonant chords [30,39] in comparison to consonant chords. In contrast, in our study, dissonant chords were presented within a musical context, which recruited subsequent higher-order processes of analysis reflected in the late neural responses (both the P3 and the N5). Once the sensory deviance of dissonance has been assessed [19], if a change in any stimulus attribute is detected, an attention-driven mechanism (reflected in the P3a) leads to further processing for updating the mental model of previous events in working memory (which is reflected in the P3b) [20,45,51]. Importantly, the larger P3 (P3a and P3b) that we observed after ending clusters suggests that they were more surprising than middle clusters, at least in trained listeners. In line with this, mistuned chords are rated worse as the tonality gets more established, although that is not reflected in an increase in the early neural responses (such as the MMN; [63]. Moreover, the lack of reduction in the P3 components suggests that, even if clusters became highly predictable through repetition, that did not render them less surprising, likely because they are strongly disruptive as a cadence closure.

### Effects of musical training

In a previous study, musicians displayed an increased EN for dissonant endings in comparison to Neapolitan endings [33]. However, it remained unclear whether such enhancement reflected the reaction to the greater sensory dissonance or the cognitive processing of clusters as stronger violation of musical context than out-of-key chords. In the present study, we replicated these results and clarified that musical-syntactic hierarchies do not influence such early processing of dissonance, as reflected by the lack of difference in the EN across positions of the cadence. Thus, the increase of the EN that we observed in musicians might reflect their more efficient processing of dissonant chords [30,32,40,53], and maybe their classification as non-prototypical chords [30]. Meanwhile, non-musicians might have more difficulty in processing the roughness of dissonance at early latencies [54] and only distinguish more conventional distinctions, such as mistuning [30].

Our results also show that musical expertise is linked to an enhancement of the P3 response toward dissonant clusters. Although we lack direct behavioral measurements of attention allocation to dissonant clusters, previous studies suggest that musicians possess faster involuntary attention allocation than non-musicians (as reflected by the P3a [2,49]). Moreover, musicians in our study showed a larger P3b, which is linked to processes of structural analysis in rule-governed sequences arising from the revision of the stimulus into the current model of the musical structure [49,50] together with the coordination of behavioral responses. That might suggest that musicians possess a more efficient updating of the sensory model [49]. The observed P3 subcomponents interacted with syntactic hierarchies suggesting that even if the early automatic detection of is independent of musical rules, musicians are still able to efficiently sort its unexpectedness according to the musical context. In short, dissonant chords might represent a stronger surprise to musicians, especially when it is placed as a cadence closure. Meanwhile, we observed an EN and a N5 in non-musicians, but neither a P3a nor a P3b. That suggests that non-musicians may have detected clusters as dissonant deviants and even attempted to integrate them, but that these clusters did not represent such a strong surprise as they did in musicians.

## Conclusion

Our study focused on the influence that syntactic (schematic) and experimental (veridical) regularities have on how the brain reacts to a purely acoustic violation such as strong dissonance. Our results show that, at early latencies, acoustic deviance may be processed independently of schematic and veridical expectations. They also show that musical training modulates the neural responses to unexpected dissonance by enhancing and refining them. Musicians showed larger responses related to the detection of dissonance (EN) and the attraction of attention (P3a), suggesting that dissonance in a musical context are more surprising for musicians. At late latencies, the neural responses of musicians reflective of attention allocation (P3a) and model-updating (P3b) were indeed influenced by syntactic (but not experimental) regularities. Moreover, while it is likely that the P3 effects overrode the N5 in musicians, in non-musicians the presence of the N5 suggested that dissonance engaged mechanisms of integration. Thus, our study advances the understanding of the processing of music by exploring the factors that modulate the neural responses to acoustic violations in a musical context and contributes to untangling the neural mechanisms behind the processing of syntactic irregularities and acoustic disruptions.

## Supporting information

S1 Data (XLS)

S2 Data (XLS)
